# Study of Serum Apelin and Insulin Resistance in Type 2 Diabetes Mellitus Patients With or Without Obesity

**DOI:** 10.7759/cureus.43401

**Published:** 2023-08-13

**Authors:** Chinmaya Mund, Chetan K Kellellu, Roma Rattan, Srikrushna Mahapatra, Andrew A Lamare, Sudeep Jena

**Affiliations:** 1 Department of Biochemistry, Government Medical College and Hospital, Sundargarh, Sundargarh, IND; 2 Department of Biochemistry, Shri Ram Chandra Bhanja (SCB) Medical College, Cuttack, IND

**Keywords:** hyperglycemia, obesity, insulin resistance, diabetes mellitus, apelin

## Abstract

Introduction: Type 2 diabetes mellitus (T2DM) is a chronic disorder characterized by persistent hyperglycemia. Chronic hyperglycemia in diabetes mellitus can cause microvascular and macrovascular complications. Obesity is a major risk factor contributing to disease progression and complications of T2DM. Apelin is an adipokine having a compensatory role in reducing insulin resistance (IR) in morbidly obese individuals. This study was undertaken to find a correlation between Apelin, IR, and obesity.

Methods: This case-control study included 180 participants, cases (n=90) having T2DM, and healthy controls (n=90). Further, the case and control groups were divided into group I (non-obese) and group II (obese) according to their body mass index (BMI) as per the Asia Pacific classification of BMI. Following obtaining consent, anthropometrical measurements and blood parameters like serum total lipid profile, fasting and postprandial glucose level, Apelin, and insulin were done, and results were analyzed statistically using Statistical Package for the Social Sciences (SPSS) version 21 (IBM Corp., Armonk, NY).

Results: A significantly higher Apelin level was observed in diabetes patients with obesity (265.16±11.0 pg/mL) as compared to non-obese (206.44±83.0 pg/mL). A positive correlation between serum Apelin levels and BMI was found (r=0.367, p=0.003). Homeostasis Model Assessment of IR (HOMA-IR) is increased in obese patients in comparison to the control group. A significant positive correlation between BMI and HOMA-IR (r=0.429, p=0.001) and Apelin and IR (r=0.742, p=0.000) was found in this study.

Conclusion: On the basis of the finding of this study, we may conclude that Apelin has a role in improving insulin sensitivity in T2DM. Larger and multicentric studies are further required to discover the therapeutic role of Apelin in T2DM.

## Introduction

Type 2 diabetes mellitus (T2DM) is a multi-factorial endocrine disorder characterized by alteration in insulin secretion, the presence of insulin resistance (IR), and a compensatory increase in hepatic gluconeogenesis. According to the Inter-National Diabetes Federation (IDF), currently, T2DM affects more than 537 million people worldwide, and by the year 2045, the number could go up to more than 783 million [1]. On the other hand, a body mass index (BMI) of over 25 is considered overweight, and a BMI of over 30 is deemed obese. According to the WHO, the epidemic of obesity has reached a critical point, with more than four million people per year dying because of being overweight or obese in 2017. Obesity rates in both adults and children are rising. 11% of males and 15% of women overall were obese [2]. According to current studies, obese people are up to 80 times more likely to get type 2 diabetes than those with a BMI of less than 22, which is considered to account for 80%-85% of the risk of developing the disease [3]. It has further been proposed that India will be home to the highest number of diabetes mellitus patients, i.e., about 80 million by the year 2030 [4]. IR is defined as a diminution in a cell, tissue, or organism’s ability to take up glucose in response to insulin. The homeostasis model of assessment (HOMA) was developed by Mathews et al. in 1985 [5]. It is a method used to quantify IR and beta cell function from basal glucose and insulin concentration.

According to the World Health Organization (WHO) overweight and obesity are defined as “abnormal or excess fat accumulation that presents a risk to health” [2]. A crude population measure of obesity is the BMI. It has been observed that obese people are more prone to IR [6]. Apelin is a recently discovered endogenous peptide hormone, released from adipocytes. Apelin is a ligand for the Apelin receptor (APJ receptor), which is a G-protein-coupled receptor (GPCR) acting via Gi/Go activation. Activation of this pathway acts through phosphoinositide-3-kinase-protein kinase B/AKT (AKT/Pi3) signaling pathway to improve glucose utilization. It has been shown that Apelin level goes up in IR patients suffering from type 2 DM. High values seen in morbidly obese T2DM cases perhaps indicating a compensatory role of Apelin in reduction of IR [7]. The studies on Apelin and its relation to IR and obesity in T2DM is recent and very sparse. There are no studies on Apelin from this part of the country. Hence, this study is undertaken to find a correlation between serum Apelin and HOMA-IR, which is a measure of IR.

## Materials and methods

Materials

This case-control study was undertaken in the postgraduate department of Biochemistry in collaboration with the Department of Medicine and Multidisciplinary Research Unit (MRU), S.C.B Medical College and Hospital, Cuttack comprising 90 samples of cases and equal numbers of matched healthy controls. The study was approved by Institutional Ethics Committee S.C.B. Medical College bearing the number 708/28.09.2018. The case group comprises patients with T2DM attending the medicine OPD. T2DM patients were enrolled based on their clinical history and biochemical parameters satisfying the criteria of the American diabetes association. Further, the case and control groups were divided into group I (non-obese) and group II (obese) according to their BMI as per the Asia Pacific classification of BMI.

Methods

All the study participants were appraised regarding the research design and after obtaining due consent, detailed demographic and anthropometric data such as BMI, diet, and therapeutic and drug history were collected. After a 12-hour overnight fasting, blood samples were collected for assay of biochemical and special parameters.

The blood glucose level was estimated by glucose oxidase peroxidase method spectrophotometrically by system pack kits adapted to Toshiba 120 FR auto analyzer. The fasting serum cholesterol level was estimated enzymatically. The cholesterol esters were hydrolyzed by the Esterase enzyme to free cholesterol and fatty acids. The free cholesterol is then oxidized to cholesterol-4-ene-3-one and hydrogen peroxide which is measured by oxidative coupling of phenol and 4-aminoantipyrine reaction by the enzyme peroxidase. The serum triglyceride level is measured by the Glycerol-3-phosphate oxidase and peroxidase coupling method. Serum high-density lipoprotein cholesterol was measured spectrophotometrically by the accelerator selective detergent method. Serum low-density lipoprotein is measured by direct method using selective detergents and enzymes such as cholesterol esterase, oxidase, and peroxidase. The serum very low-density cholesterol was calculated by the Fried Wald formula. The biological reference range was as per the guidelines of the National Cholesterol Education program. The kidney function was assessed by serum urea, creatinine, and electrolytes level. The electrolytes were measured by Ion selective electrode method. Serum urea and creatinine were enzymatically estimated by urease and creatinine kinase methods respectively. The serum fasting Insulin was evaluated by electrochemiluminescence method by automated ECLIA e411 Cobas model from Roche Diagnostics. The serum Apelin was estimated by Enzyme Linked Immuno Assay by automated ELISA reader and washer from Bio-rad company.

Statistical analysis

All the numerical data is represented as mean ± SD. Unpaired students t-test, One-way ANOVA, and post hoc Tuckey Honestly Significant Difference (HSD) were used to test the statistical significance of the data. A p-value less than 0.05 was considered significant. The correlation of parameters was measured by Pearson's correlation assay, logistic regression, and multivariate analysis. The statistical data analysis was done using SPSS version 21 software (IBM Corp., Armonk, NY).

## Results

The demographic profile comparison shows that waist circumference (WC), BMI, and weight of the individuals are directly associated with the incidence of disease. A p-value of 0.001 suggests a strong positive association between WC and T2DM (Table 1).

**Table 1:** Comparison of demographic profile among study group

Parameters	Healthy Volunteers (Mean±S.D.) (N=90)	T2DM Patients (Mean±S.D.) (N=90)	P-value
Age (years)	39.28±8.9	46.64±11.2	0.431
Height (cm)	157±9.6	163±11.4	0.190
Weight (kg)	64.9±9.4	72.2±11.6	0.035*
BMI (kg/m^2^)	23.4±3.6	28.54±3.9	0.037^*^
WC (cm)	81.2±10.2	88.6±9.7	0.001^*^

Total cholesterol and triglyceride levels were found to be elevated among the T2DM patients in our study whereas the HDL levels are higher for control groups (Table 2).

**Table 2:** Comparison of lipid profile among study groups

Parameters	Healthy Volunteers (Mean±S.D.) (N=90)	T2DM Patients (Mean±S.D.) (N=90)	P-value
Total Cholesterol (mg/dL)	169.4±36.75	221.5±35.17	0.547
Triglycerides (mg/dL)	131.42±65.4	169.44±84.3	0.051*
High Density Lipoprotein Cholesterol (mg/dL)	52.63±8.32	47.21±7.29	0.002*
Low Density Lipoprotein Cholesterol (mg/dL)	93.51±34.26	124.87±40.36	0.190
Very Low Density Lipoprotein Cholesterol (mg/dL)	26.46±12.86	29.72±13.45	0.021*

BMI among the study groups is done by using one-way ANOVA test. It shows a higher BMI among the case groups as compared to the control (Table 3).

**Table 3:** Comparison of BMI among study groups All data were represented by Mean ± SD and were compared by one-way ANOVA ^a^Statistically significant as compared to control group I and control group II (p=0.000) ^b^Statistically significant as compared to case group I and control group I (p=0.017) ^c^Statistically significant as compared to case group I and case group II (p=0.001)

Parameters	Control Group I (Mean± S.D.) (N=45)	Control Group II (Mean±S.D.) (N=45)	Case Group I (Mean± S.D.) (N=45)	Case Group II (Mean±S.D.) (N=45)
BMI (kg/m^2^)	21.32±2.7	26.77±3.7^a^	23.45±1.7^b^	28.31±2.5^c^

HOMA IR comparison between study groups demonstrates the significance of IR. Control groups have lower HOMA IR values as compared to case groups. A very high HOMA IR value (7.9±2.4) is seen in obese T2DM patients (Table 4).

**Table 4:** Comparison of HOMA IR among study groups All data were represented by Mean ± SD and were compared by one-way ANOVA ^a^Statistically significant as compared to control group I and control group II (p=0.001) ^b^Statistically significant as compared to case group II with control group I (p=0.002) ^c^Statistically significant as compared to case group I and case group II (p=0.000)

Parameters	Control Group I (Mean±S.D.) (N=45)	Control Group II (Mean±S.D.) (N=45)	Case Group I (Mean±S.D.) (N=45)	Case Group I (Mean±S.D.) (N=45)
HOMA IR	1.9±1.1	3.8±0.8^a^	4.2±1.6^b^	7.9±2.4^c, b^

Higher Apelin value was observed in case groups than control groups. We also observed a significantly higher Apelin level in obese cases (265.16±11.0 pg/mL) when compared to non-obese (206.44±83.0 pg/mL) (Table 5).

**Table 5:** Comparison of serum APELIN among study groups All data were represented by Mean ± SD and were compared by one-way ANOVA ^a^Statistically significant as compared to control group I and case group I (p=0.034) ^b^Statistically significant as compared to control group II and case group II (p=0.001) ^c^Statistically significant as compared to case group I and case group II (p=0.000)

Parameters	Control Group I (Mean± S.D.) (N=45)	Control Group II (Mean±S.D.) (N=45)	Case Group I (Mean± S.D.) (N=45)	Case Group II (Mean±S.D.) (N=45)
APELIN (pg/mL)	117.98±15^a^	148.24±21^b^	206.44±83^c^	265.16±110

**Figure 1:**
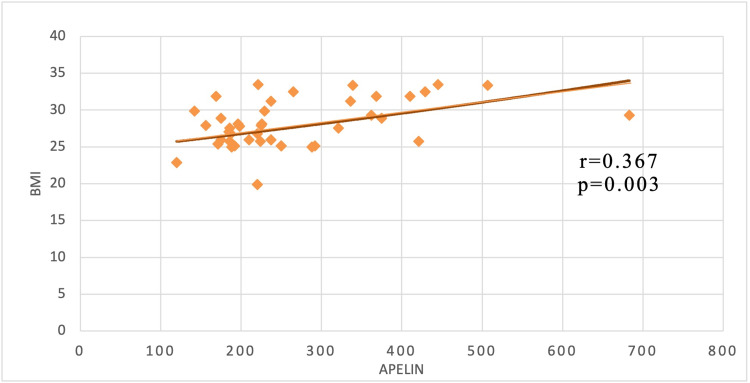
Correlation between serum apelin and BMI in case groups

Pearson’s correlation analysis between serum Apelin (pg/mL) levels and BMI shows a positive correlation with a r-value of 0.367 and a p-value of 0.003 (Figure 1).

**Figure 2:**
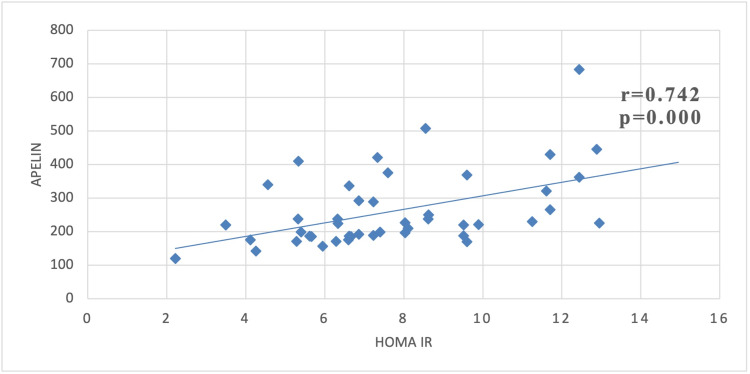
Correlation between Apelin and HOMA IR in case groups

Pearson's correlation analysis between serum Apelin (pg/mL) and IR in the form of HOMA IR. A significant positive correlation was observed with a r-value of 0.742 and a p-value of 0.000 (Figure 2).

**Figure 3:**
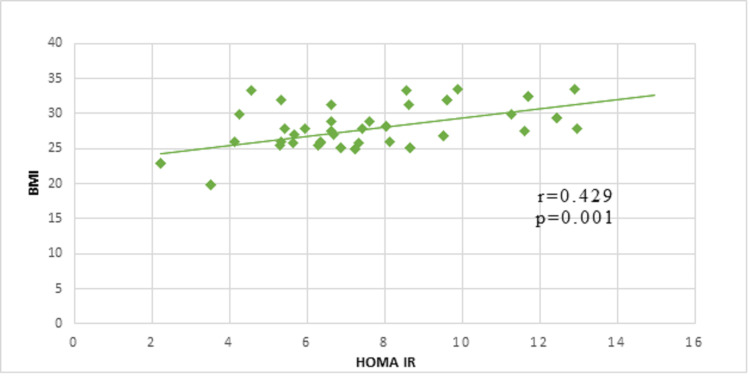
Correlation between BMI and HOMA IR in case groups

Pearson’s correlation analysis between BMI and HOMA IR. A significant positive correlation was observed with a r-value of 0.429 and a p-value of 0.001 (Figure 3).

## Discussion

Diabetes mellitus is a group of metabolic disorders characterized by hyperglycemia, resulting from defects in insulin secretion, insulin action, or both. India is among the top three countries with a high diabetic population in the year 2016. It has further been proposed that India will be home to the highest number of diabetes mellitus patients, i.e., about 80 million by the year 2030 [4].

Obesity is regarded as the cause of various lifestyle diseases such as hypertension, metabolic syndrome, IR, maturity-onset T2DM, and coronary artery disease (CAD) [8]. WHO has recognized both obesity and diabetes mellitus as epidemic diseases in the 21st century [2,9]. Various research works implicate the role of truncal and visceral obesity in producing non-communicable lifestyle diseases such as hypertension, diabetes mellitus, and dyslipidemia [10].

 The adipose cell produces several types of cytokines known as adipokines which influence endocrine signaling pathways such as regulation of insulin signaling in various tissues in the body and may thus provide a molecular link between obesity and T2DM. Adipokines have a major role in obesity-related co-morbidities and complications [11]. Studies by Stepien et al. have shown that disturbances of adipokines secretion may contribute to peripheral IR [12]. Apelin is a recently discovered endogenous peptide hormone that has garnered significant investigative attention during the last decade. Apelin is a G-protein-coupled receptor (APJ) ligand as well as a regulatory peptide with wide distribution in the human body. The apelin-APJ system has attracted increasing attention over the past few years because of its possible involvement in a number of physiological processes, including glucose and lipid metabolism. Apelin is crucial for controlling insulin secretion and plays a role in the pathogenesis of diabetes complications [13].

Our study evaluated the data of obese T2DM patients and healthy volunteers with lean T2DM patients to investigate the role of Apelin and obesity in contributing to the development of IR, CAD, and impaired glycemia. In this study, we found that there is a significant difference in height, weight, BMI, and WC among the cases and controls, respectively (p<0.05) (Table 1). We observed a significant difference in serum TG and HDL-C among the cases and controls, respectively (p<0.05). These results agree with Ba et al. [14]. But there is no significant difference in total cholesterol and LDL-C among cases and controls in our study.

The serum Apelin level was significantly increased in obese T2DM patients as compared to lean patients and healthy volunteers. A similar finding was observed in the study of Boucher et al., which stated that apelin is primarily produced and secreted by adipose tissue. Therefore, an increase in the volume of Adipose tissue leads to a proportional rise in the production of Apelin and the expression of the Apelin receptor [15].

In our study, a positive significant correlation was observed between Apelin and BMI, which implies its origin from adipocytes in response to metabolic and endocrine alteration. This finding is in agreement with the study conducted by Sheibani et al. [16]. Thus, our study rationalizes that serum Apelin can be used as a marker particle having endocrine potency to predict metabolic and nutritional changes. Various recent studies associated the serum Apelin level with fluctuation in body weight and obesity [17].

Also, in our study a significant positive correlation between Apelin and IR is found which is also supported by the works of Boucher et al. who suggested an impaired Apelin homeostasis can be resulted from increased insulin concentration, which ultimately increases Apelin level [15]. Also, there is a potent correlation between HOMA-IR and BMI which is in agreement with the study of Li et al. [18]. Some studies have shown that Apelin suppresses the secretion of insulin plasma systems and also Apelin modulates lipolysis and fatty acid oxidation, promotes glucose absorption, and improves insulin sensitivity. Administering apelin can help to treat the complications associated with diabetes. As a result, diabetes and its related complications could be treated by targeting the apelin-APJ system [13]. It was found that administering exogenous apelin improved glucose metabolism [19]. On top of that, both type 2 diabetic and normal isolated adipocytes showed apelin-induced glucose absorption [19,20]. These findings suggest that apelin may function under conditions of high insulinemia as an exogenous insulin sensitizer. Diabetic patients' pancreatic islet mass and insulin levels are significantly improved by long-term apelin treatment [21]. Due to its capacity to improve insulin sensitivity, many authors also hypothesized that Apelin may operate as a potent insulin-sensitizing factor and could be a useful target for management [22].

## Conclusions

Raised serum Apelin in T2DM may be a compensatory mechanism to the IR state found in these patients. Hyperinsulinemia seen in T2DM stimulates Apelin secretion from adipocytes, which in turn facilitates glucose absorption by Gi-, Gq- and AMP-activated protein kinase (AMPK) mediated pathways. Therefore, Apelin seems to be an intricate part of the compensatory mechanism of insulin sensitivity. But the exact mechanism of its action is still yet to be elucidated.

On the basis of the finding of this study, we can conclude that Apelin has a role in improving insulin sensitivity in T2DM, but the available research on the therapeutic role of Apelin remains unrevealed as of date. However, larger and multicentric studies may elucidate the therapeutic role of Apelin in T2DM.
